# Patients with psychological ICPC codes in primary care; a case-control study investigating the decade before presenting with problems

**DOI:** 10.1080/13814788.2017.1359536

**Published:** 2017-09-15

**Authors:** Luc G. Gidding, Mark G. Spigt, Geert-Jan Dinant

**Affiliations:** ^a^ Research Institute CAPHRI, Department of Family Medicine, Maastricht University Maastricht The Netherlands; ^b^ Department of Community Medicine, Tromso University Tromso Norway; ^c^ Department of Public Health, Mekelle University Mekelle Ethiopia

**Keywords:** Psychological problems, epidemiology, case-control designs, anxiety, depression, somatization, surmenage, sleep

## Abstract

**Background:** Recognizing patients with psychological problems can be difficult for general practitioners (GPs). Use of information collected in electronic medical records (EMR) could facilitate recognition.

**Objectives:** To assess relevant EMR parameters in the decade before patients present with psychological problems.

**Methods:** Exploratory case-control study assessing EMR parameters of 58 228 patients recorded between 2013 and 2015 by 54 GPs. We compared EMR parameters recorded before 2014 of patients who presented with psychological problems in 2014 with those who did not.

**Results:** In 2014, 2406 patients presented with psychological problems. Logistic regression analyses indicated that having registrations of the following statistically significant parameters increased the chances of presenting with psychological problems in 2014: prior administration of a depression severity questionnaire (odds ratio (OR): 3.3); fatigue/sleeping (OR: 1.6), neurological (OR: 1.5), rheumatic (OR: 1.5) and substance abuse problems (OR: 1.5); prescriptions of opioids (OR: 1.3), antimigraine preparations (OR: 1.5), antipsychotics (OR: 1.7), anxiolytics (OR: 1.4), hypnotics and sedatives (OR: 1.4), antidepressants (OR: 1.7), and antidementia drugs (OR: 2.1); treatment with minimal interventions (OR: 2.2) and physical exercise (OR: 3.3), referrals to psychology (OR: 1.5), psychiatry (OR: 1.6), and psychosocial care (OR: 2.1); double consultations (OR: 1.2), telephone consultations (OR: 1.1), and home visits (OR: 1.1).

**Conclusion:** This study demonstrates that possible indications of psychological problems can be identified in EMR. Many EMR parameters of patients presenting with psychological problems were different compared with patients who did not.

KEY MESSAGES

- General practitioners can have problems with recognizing psychological problems.
- The use of electronic medical records (EMR) data could be an interesting option for timely recognition.
- Patients who presented with psychological problems showed a different medical history than patients who did not. These results could help GPs be more attentive to possible indications of psychological problems.

## Introduction

Patients who suffer from psychological problems generate a significant burden on healthcare systems, especially primary care [1]. This burden consists of massive healthcare consumption and high costs [2,3]. Patients present with psychological symptoms at a later time and less straightforward than most somatic symptoms, which induces suboptimal recognition of psychological problems in primary care [4,5]. Many general practitioners (GPs) admit struggling with management of psychological problems [6,7].

To control this healthcare burden, a considerable body of research has focussed on the optimization of diagnosis and treatment of people who present with psychological problems in primary care [8,9].

Arguably, a more proactive and preventive approach could lead to patients with less severe symptoms, better treatment outcomes and lower cost development, in primary and in secondary care since adequate treatment is cost-effective and reduces the burden of disease [10]. Early and proactive recognition and treatment of patients with psychological problems have previously shown to reduce disease onset, symptom levels and relapse, and improve treatment adherence, social behaviour, work productivity and absenteeism [11]. In this approach, patients should be identified as cases of interest before actually presenting with problems. Routine screening of all patients in primary care for psychological problems using questionnaires seems neither feasible nor desirable [12]. The use of data already routinely collected in electronic medical records (EMR), appears a more interesting option [13].

The identification of EMR parameters useful for such an approach first requires a better understanding of the period before patients present with psychological problems. Therefore, we assessed relevant EMR parameters in a large group of primary care patients during the decade before presenting with problems.

## Methods

### Design

An exploratory case-control study assessing relevant EMR parameters of a population of 58 228 patients recorded between 2003 and 2015 by 54 GPs. We compared the EMR parameters recorded before 2014 of patients who presented with relevant psychological problems in 2014 with those who did not.

### Setting

The Eindhoven Corporation of Primary Health Care Centres (Stichting Gezondheidscentra Eindhoven (SGE)), which includes 10 multidisciplinary primary healthcare centres in Eindhoven, the Netherlands. During the study period, 54 different GPs were employed, ranging from 38 in 2003 to 47 in 2014.

### Data collection

Since 2003, these healthcare centres used the same digital information system for their EMR (Medicom, Pharmapartners, Oosterhout). Data were extracted and analysed in IBM SPSS Statistics for all patients of all ages registered at any time in the study period from July 2003 until January 2015. All patient information was extracted anonymously and only regarding patients who had previously given their permission for the analyses of their EMR for research purposes. Regarding data quality, our previous publications pertaining to this setting showed high registration quality [14,15].

### Identification of two groups of patients

We identified patients presenting with relevant psychological problems as those patients for whom the GPs registered one or more of the following psychological codes from the international classification of primary care (ICPC-1) as reason for encounter in 2014: feeling anxious (P01), acute stress reaction (P02), post-traumatic stress disorder (P02.01), feeling depressed (P03), feeling angry (P04), other organic psychosis (P71), delirium (P71.04), schizophrenia (P72), affective psychosis (P73), bipolar disorder (P73.02), anxiety disorder (P74), panic disorder (P74.01), generalized anxiety disorder (P74.02), hypochondria (P75), depression (P76), postpartum depression (P76.01), suicide attempt (P77), suicide attempt (P77.01), suicide (P77.02), neurasthenia/surmenage (P78) mental exhaustion, neurosis (P79), phobia (P79.01), obsessive-compulsive disorder (P79.02), personality disorder (P80), borderline personality disorder (P80.01), other psychosis (P98) and burnout (Z29.01); and named these ‘P-cases.’ The group of ‘non-P-cases’ consisted of those who were registered patients at any time in 2014 but did not receive one or more of these ICPC codes as reason for encounter in 2014. From these two groups, we excluded 12 311 patients who had one or more of these ICPC codes before 2014.

### Relevant care parameters

For both groups, we identified demographics, administrations of the 4-dimensional symptom questionnaire (4DSQ) and Beck depression inventory-2 (BDI2), previous morbidity (Supplementary Table 1), pharmacotherapy, other treatments, referrals, and consumption of GP services [16].

**Table 1.** Demographics.

	P-cases *n* = 2406 (4.1%)	Non-P-cases *n* = 55 822 (95.9%)	*p*-value
Female	60.7%	48.1%	<.05
Median age 1 January 2015 (IQR)	38.9 (27.8–55.3)	36.6 (18.6–57.2)	<.05
Median duration as registered patient (IQR)	10.5 (4.7–10.5)	9.4 (3.8–10.5)	<.05
Civil state			<.05
Married/living with partner	31.4%	31.5%	
Unmarried	23.6%	26.7%	
Divorced	3.1%	1.3%	
Widow(er)	3.0%	2.0%	
Unknown	39.0%	38.6%	
Median socio-economic status score (IQR)	−0.38 SD (−0.59 to 0.64)	−0.30 SD (−0.54 to 0.96)	<.05

IQR: interquartile range.

Demographics included gender, age on 1 January 2015, duration as registered patients between July 2003 and January 2014, civil status and socio-economic status (SES). SES was based on status scores derived from postal codes by the Netherlands Institute for Social Research [17,18]. The scores were based on average income, the percentage of people with low income, the percentage of people with a lower educational level and the percentage of people that did not work, per postal code. The Netherlands Institute for Social Research calculated these scores since 1998 and the average score over the period 1998–2014 was zero. The SES scores were defined as the standard deviation (SD) surrounding the average score in 1998–2014. The average SES score in 2014 was 0.28.

All parameters hereafter have been assessed for the period 1 July 2003 until 1 January 2014.

The 4DSQ is a patient questionnaire that assesses distress, anxiety, depression and somatization and was recommended in the Dutch primary care guidelines for anxiety and depression [16,19–21]. The BDI2 is a psychometric questionnaire to measure the severity of depression and has been used as a required monitoring tool for depression treatment progress in this setting since 2008 [14,22]. For both questionnaires, we calculated the number of administrations per patient and the median scores per scale for all administrations.

To measure previous morbidity, we grouped relevant ICPC-1 codes into clusters of conditions (Supplementary Table 1). A patient was defined as suffering from a condition cluster when a patient had one or more registrations of ICPC codes from such a cluster between July 2003 and January 2014. We calculated the number of ICPC episodes and median episode duration for all patients.

Prescriptions for medication by the GPs were analysed for all subgroups of the Anatomical Therapeutic Chemical (ATC) Classification System ‘nervous system’ (‘N’) chapter. Prescriptions were defined as separate when there were more than seven days between them. The median number of prescriptions and days per prescription were calculated for all patients.

Relevant non-pharmacological treatment and referral options included dietary care, minimal interventions, physical exercise, psychology, psychiatry and psychosocial care. The minimal interventions included brief counselling, bibliotherapy, mindfulness, psycho-education, problem-solving therapy, or referral to a social worker. Physical exercise therapies involved running therapy, physiotherapy, manual therapy and Cesar therapy. Psychosocial care consisted of clinics for alcohol and drugs counselling, complex psychosocial care, regional institutes for outpatient mental healthcare, psychogeriatrics, and social psychiatric nurses. The median number of referrals was calculated for all patients.

Consumption of GP services was calculated by totalling the number of office consultations with GPs or practice nurses, telephone consultations, and home visits for all patients.

### Statistical analysis

All medians were calculated based on the patients who had a registration of a specific parameter. *p*-values to assess whether the groups were significantly different were calculated with Pearson’s chi square tests and independent samples Mann–Whitney U tests.

Besides these bivariate analyses, we also performed a logistic regression analysis per table, calculating odds ratios for the separate factors in the tables in relation to the outcome; presenting with relevant psychological problems in 2014. All factors were used as categorical factors in the regressions (e.g. presence of specific morbidity or being referred before 2014, yes/no) excepting consumption of GP services, for which the continuous median consumption per year was used.

## Results

In 2014, there were 2406 (4.1%) P-cases who had an EMR registration of one or more relevant ICPC codes as reason for encounter. There were 55 822 (95.9%) non-P-cases in 2014. In the Supplementary tables (available online), we present more detailed results.

### Demographics

Table 1 shows the demographics of the two groups. The patients registered with a relevant ICPC code in 2014 were more often female and older. They also had lower SES scores. Although the groups differed significantly (*p*-value <.05) in median duration as registered patients, these medians were comparable for both groups.

### Mental health questionnaires

Of the 2406 P-cases, six patients (0.2%) were administered the 4DSQ and 28 (1.2%) the BDI2 before 2014 (Supplementary Table 2). The (sub) scores on both questionnaires were similar. In the logistic regression, administrations of both questionnaires were included as categorical factors. We found that patients who had a BDI2 administration before 2014 had significantly higher chances of presenting relevant psychological problems in 2014 (OR: 3.3, *p*-value <.05) compared with those who did not fill in a BDI2.

**Table 2.** Morbidity before 2014. The median N episodes per patient were one for all listed conditions for both groups (IQR 1–1).

			Bivariate analysis	Logistic regression^a^	
Condition clusters	P-cases *n* = 2406 *n*, (%)	Non-P-cases *n* = 55 822 *n*, (%)	*p*-value	OR (95%CI)	*p*-value
Cancer	133 (5.5)	3206 (5.7)	.656	0.9 (0.7–1.1)	.248
Cardiovascular disease	361 (15.0)	8195 (14.7)	.661	0.9 (0.8–1.0)	.075
Fatigue/sleeping disorders	386 (16.0)	5634 (10.1)	<.05	1.6 (1.4–1.8)	<.05
Irritable bowel disease	14 (0.6)	386 (0.7)	.524	0.8 (0.4–1.3)	.307
Metabolic/eating disorders	226 (9.4)	4412 (7.9)	<.05	1.1 (1.0–1.3)	.082
Neurological disorders	178 (7.4)	2616 (4.7)	<.05	1.5 (1.3–1.8)	<.05
Respiratory disorders	145 (6.0)	3012 (5.4)	.181	1.0 (0.8–1.2)	.933
Rheumatic disorders	46 (1.9)	667 (1.2)	<.05	1.5 (1.1–2.0)	<.05
Substance abuse	203 (8.4)	3133 (5.6)	<.05	1.5 (1.3–1.7)	<.05

OR: odds ratio; 95%CI: 95% confidence interval.

^a^ Logistic regression analysis with being a ‘P-case’ as dependent variable and having ≥1 ICPC codes from the separate condition clusters as dichotomous independent variables. Table 1 in the Supplementary provides an overview of the ICPC codes in the separate condition clusters.

### Previous morbidity

As to previous morbidity in Table 2, all but two condition clusters were present at higher rates in the P-cases as opposed to the non-P-cases before 2014. Episode duration did not seem to differ between the two groups (Supplementary Table 3). The median number of episodes per patient was one for all listed conditions for both groups (IQR 1–1).

**Table 3.** Nervous system medication before 2014. Unlisted ATC ‘N’ groups were not prescribed.

			Bivariate analysis	Logistic regression^b^	
ATC groups	P-cases *n* = 2406 *n* (%)	Non-P-cases *n* = 55 822 *n* (%)	*p*-value	OR (95%CI)	*p*-value
General anaesthetics (N01A)^a^	1 (0)	1 (0)	<.05		
Local anaesthetics (N01B)	136 (5.7)	2859 (5.1)	.248	0.9 (0.8–1.1)	.469
Opioids (N02A)	253 (10.5)	3549 (6.4)	<.05	1.3 (1.1–1.5)	<.05
Other analgesics and antipyretics (N02B)	243 (10.1)	4175 (7.5)	<.05	1.0 (0.9–1.2)	.903
Antimigraine preparations (N02C)	98 (4.1)	1241 (2.2)	<.05	1.5 (1.2–1.9)	<.05
Anti-epileptics (N03A)	54 (2.2)	794 (1.4)	<.05	0.9 (0.7–1.3)	.726
Anticholinergic agents (N04A)^a^	0 (0)	9 (0)	.533		
Dopaminergic agents (N04B)	18 (0.7)	184 (0.3)	<.05	1.4 (0.9–2.4)	.164
Antipsychotics (N05A)	23 (1.0)	195 (0.3)	<.05	1.7 (1.1–2.6)	<.05
Anxiolytics (N05B)	284 (11.8)	3.666 (6.6)	<.05	1.4 (1.3–1.7)	<.05
Hypnotics and sedatives (N05C)	237 (9.9)	2.944 (5.3)	<.05	1.4 (1.2–1.7)	<.05
Antidepressants (N06A)	132 (5.5)	1.245 (2.2)	<.05	1.7 (1.4–2.1)	<.05
Psychostimulants (N06B)	12 (0.5)	256 (0.5)	.776	1.0 (0.5–1.8)	.953
Antidementia drugs (N06D)	12 (0.5)	93 (0.2)	<.05	2.1 (1.1–3.9)	<.05
Parasympathomimetics (N07A)^a^	1 (0)	8 (0)	.293		
Drugs used in addictive disorders (N07B)	53 (2.2)	779 (1.4)	<.05	1.2 (0.9–1.6)	.173
Anti-vertigo preparations (N07C)	66 (2.7)	1.114 (2.0)	<.05	1.0 (0.8–1.3)	.940
Other nervous system drugs (N07X)^a^	1 (0)	1 (0)	<.05		

^a^ ATC groups were excluded from logistic regression analysis due to insufficient patients.

^b^ Logistic regression analysis with being a ‘P-case’ as dependent variable and having ≥1 prescriptions from the separate ATC groups as dichotomous independent variables.

The logistic regression analysis with all morbidity clusters presented in Table 2 included, showed that prior episodes of fatigue/sleeping, neurological, rheumatic and substance abuse problems significantly increased the chances of presentation with psychological problems in 2014.

### Pharmacotherapy

Nervous system medications were prescribed for a substantial part of the P-cases, especially opioids (11%), other analgesics and antipyretics (10%), anxiolytics (12%) and hypnotics and sedatives (10%) (Table 3). All prescribed nervous system medication groups were prescribed more often to P-cases before 2014. The differences in durations per prescription were not clinically relevant (Supplementary Table 4). The logistic regression analysis indicated that opioids (OR: 1.3), antimigraine preparations (OR: 1.5), antipsychotics (OR: 1.7), anxiolytics (OR: 1.4), and hypnotics and sedatives (OR: 1.4), antidepressants (OR: 1.7) and antidementia drugs (OR: 2.1) significantly increased the chances of presentation with psychological problems in 2014.

**Table 4.** Treatments/referrals before 2014.

			Bivariate analysis	Logistic regression^a^	
Treatments/referrals	P-cases *n* = 2406 *n* (%)	Non-P-cases *n* = 55 822 *n* (%)	*p*-value	OR (95%CI)	*p*-value
Dietary care	100 (4.2)	2042 (3.7)	.204	1.0 (0.8–1.3)	.841
Minimal interventions	128 (5.3)	1199 (2.1)	<.05	2.2 (1.8–2.7)	<.05
Physical exercise therapies	456 (19.0)	9092 (16.3)	<.05	1.1 (1.0–1.2)	<.05
Psychology	192 (8.0)	2649 (4.7)	<.05	1.5 (1.3–1.7)	<.05
Psychiatry	53 (2.2)	626 (1.1)	<.05	1.6 (1.2–2.1)	<.05
Psychosocial care	22 (0.9)	191 (0.3)	<.05	2.1 (1.4–3.3)	<.05

^a^ Logistic regression analysis with being a ‘P-case’ as dependent variable and having ≥1 of the separate treatments/referrals as dichotomous independent variables. The separate treatments/referrals are explained in the methods.

### Other treatments and referrals

The P-cases were referred at statistically significant higher rates to minimal interventions, physical exercise, psychology, psychiatry and psychosocial care (Table 4). A prior referral to one of these also significantly increased the chances of presenting with a psychological problem in 2014.

### Consumption of GP services

Table 5 shows that fewer P-cases had consultations with their GPs than non-P-cases (69% versus 80%) and less had telephone consultations (61% versus 64%). Yet, they consumed the other types of healthcare activities more often. Although the rates of consumption of GP services were quite similar, the P-cases seemed to consume more of all care types per year. The logistic regression analysis showed that having one more consultation with the GP decreased the chance of presentation with psychological problems in 2014 (OR: 0.96 *p*-value <.05). Consuming more double consultations, telephone consultations or home visits significantly increased these chances, although these odds ratios remained small (1.19, 1.06 and 1.14, respectively).

**Table 5.** Consumption of GP services before 2014.

			Bivariate analysis	Logistic regression^a^	
Types of consumption	P-cases *n* = 2406	Non-P-cases *n* = 55 822	*p*-value	OR (95%CI)	*p*-value
Consultations	*n* = 1650 (68.6%)	*n* = 44 362 (79.5%)	<.05		
Median N per patient per year (IQR)	2 (2–4)	2 (1–3)	<.05	0.96 (0.93–0.99)	<.05
Double consultations	*n* = 1386 (57.6%)	*n* = 30 521 (54.7%)	<.05		
Median N per patient per year (IQR)	1 (1–2)	1 (1–2)	<.05	1.19 (1.14–1.24)	<.05
Telephone consultations	*n* = 1460 (60.7%)	*n* = 35 559 (63.7%)	<.05		
Median N per patient per year (IQR)	2 (1–2)	1 (1–2)	<.05	1.06 (1.02–1.10)	<.05
Home visits	*n* = 215 (8.9%)	*n* = 3984 (7.1%)	<.05		
Median N per patient per year (IQR)	1 (1–2)	1 (1–1)	<.05	1.14 (1.06–1.23)	<.05

^a^ Logistic regression analysis with being a ‘*p*-case’ as dependent variable and the median N per patient per year as continuous independent variables.

## Discussion

### Main findings

Patients who presented with psychological problems (i.e. ‘P-cases’) in 2014 showed significantly higher rates of BDI2 administrations, episodes of fatigue/sleeping, metabolic/eating, neurological, rheumatic and substance abuse problems, several nervous system medication group prescriptions, all treatment and referral options except dietary care, double consultations and home visits before 2014 compared with the non-P-cases.

### Interpretation of findings

#### Demographics

The median durations as registered patients were vast and comparable for both groups (9–10 years). This enhanced comparability of both groups i.e. the windows of opportunity in which GPs could register parameters of interest was equal.

#### Previous morbidity

Our study produced parameters that significantly increased the chances of presenting with relevant psychological problems in 2014. Concerning previous morbidity, especially patients with fatigue/sleeping, neurological, rheumatic and substance abuse problems should warrant additional awareness from GPs of (future) psychological problems in patients presenting with these morbidities, which have already shown to increase the risk of anxiety and depression, for example [23,24].

#### Pharmacotherapy and other treatments and referrals

Many of the medication groups and treatment and referral options showed increased risk of presentation with psychological problems, which could be because these were psychology oriented and could be seen as preliminary actions leading up to a relevant ICPC registration. A more in-depth analysis of prescribed medication and treatments is possible based on the EMR used in this setting, i.e. including all ATC groups. For this study, we chose only to assess psycho-medication because of the number of results we already presented. Broadening the ATC scope would be interesting and enhance the comparability of this setting with other studies on medication associated with psychological problems [25].

Additionally, the P-cases were referred more often to physical exercise therapies, possibly indicating that the P-cases also had more lifestyle-orientated problems in their medical history.

#### Consumption of GP services

Besides the specific medical history of P-cases, they consumed more double consultations and home visits than non-P-cases. The P-cases also showed higher numbers in total and per year. Frequent re-attendance is an established predictor of psychological problems [2,26]. In our results, this is reflected in the bivariate analyses indicating higher numbers of all types of consumption of GP services for the P-cases, both in total as well as per year. Hence, it might seem odd that in the logistic regression analysis, a higher number of consultations per year was associated with a decreased chance of presenting with psychological problems (OR: 0.96, *p*-value <.05). However, this is compensated by the results of the logistic regression analysis concerning the other types of consumption of GP services. This is an indication that P-cases had a different care pattern, seeing the GP more often and longer per contact moment.

### Strengths and limitations

This study can be useful in showing the diversity and usefulness of information incorporated in EMR. EMR can provide clinical information of many patients over an extended period, providing means to assess usual care in everyday practice, without the workload and drawbacks of research projects in which care providers and patients actively have to participate. Although EMR can provide relevant data, some data that would have been very interesting were missing in our study. This is a difference between EMR research and patient questionnaire research. For instance, social and personal circumstances of the patients would have been interesting and valuable, but were lacking in our data. Previous research disclosed several socio-economic factors that influenced the occurrence of psychological problems, such as a civil state, SES, functional impairment, social contacts, life events, bereavement, ethnicity [23,24,27–29]. In the EMR we assessed, (changes in) such factors were under registered or difficult to extract. Indeed, we included civil state and SES, but the civil state of a significant percentage of patients was unknown and SES was a proxy based on postal codes. This could be potentially solved by more registration of such factors in EMR or combining questionnaires and EMR assessments in future research on predictors. This could contribute to reducing the problem that few studies on predictors and risk factors for psychological problems assess the same factors [24].

Factors that possibly predict the occurrence of psychological problems have been described before including female gender, lower SES, chronic conditions, sleeping problems and disability, though to our knowledge this is the first study to present different usable parameters derived from EMR from one healthcare system [23,24,30]. Also, our results indicate that predictors, previously found in research actively involving patients, can be found in EMR. Hypothetically, other primary care practices could use our methods to disclose relevant local EMR parameters with predictive value concerning their particular population of patients, to target their high-risk groups more specifically.

### Implications for practice

The large number of EMR parameters statistically significantly associated with psychological problems indicates that it is probably not possible to identify one or two specific predictors but that it is much more a palette of comorbidity, use of medication and other therapies, and frequent attendance that should induce increased alertness from GPs and other primary care providers. Moreover, this palette can be observed when reviewing the EMR.

Most of the associated parameters we found were related to psychological problems. More unexpected, non-psychological parameters included fatigue/sleeping, neurological, rheumatic and substance abuse problems, and physical exercise therapy.

Based on the presented parameters, primary care providers could be supported in identifying patients prone to present psychological problems in the future. These patients could then be invited for a GP consultation or offered a psychometric questionnaire in the case of diagnostic uncertainty or to help the patient gain insight.

## Conclusions

This study demonstrates that possible indications of psychological problems can be identified in EMR. Patients who presented with psychological problems to their GPs in 2014, showed large differences in their EMR parameters in the decade before compared with those who did not present with these problems.

## Supplementary Material

Supplemental Tables 1-6
