# Supplementary material for: Patients with psychological ICPC codes in primary care; a case-control study investigating the decade before presenting with problems
Source: Eur J Gen Pract. 2017 Sep 15;23(1):217–24. doi: 10.1080/13814788.2017.1359536 (PMC5774286; doi:10.1080/13814788.2017.1359536)
Supplement: Supplemental Tables 1-6 [file IGEN_A_1359536_SM8509.docx]

Table 1. Description of condition clusters

| **Condition clusters** | **ICPC codes** |
| --- | --- |
| Cancer | A79, B72, B72.01, B72.02, B73, B74, B74.01, D74, D75, D76, D77, D77.01, D77.02, D77.03, D77.04, F74.01, H75.01, K72.01, L71.01, N74, R84, R85, S77, S77.01, S77.02, S77.03, S77.04, T71, U75, U76, U77, X75, X76, X76.01, X77, X77.01, X77.02, Y77, Y78, Y78.01, Y78.02, Y78.03, Y79.03 |
| IBD | D94, D94.01, D94.02 |
| Cardiovascular | K74, K74.01, K74.02, K75, K76, K76.01, K76.02, K77, K77.01, K77.02, K78, K86, K87, K89, K90, K90.01, K90.02, K90.03 |
| Rheumatic | L18.01, L88, L88.01, L88.02 |
| Neurological | N86, N87, N87.01, N88, N89, P70, P70.01, P70.02 |
| Anxiety | P01, P74, P74.01, P74.02, P79, P79.01, P79.02 |
| Depression | P03, P76, P76.01 |
| Substance abuse | P15, P15.01, P15.02, P15.03, P15.05, P15.06, P17, P18, P19, P19.01, P19.02, |
| Fatigue/ sleeping | A04, A04.01, P06, P06.01 |
| Stress/ agitation | P02, P02.01, P04, P75, P78, Z29.01 |
| Suicidal ideation/ suicide | P77, P77.01, P77.02 |
| Personality | P80, P80.01 |
| Psychotic | P71, P71.04, P72, P73, P73.02, P98 |
| Respiratory | R91, R91.01, R91.02, R95, R96 |
| Metabolic/ eating | T06, T06.01, T06.02, T82, T83, T90, T90.01, T90.02 |

Table 2. Use of questionnaires before 2014

|  |  |  | **Bivariate analysis** | **Logistic regression†** | |
| --- | --- | --- | --- | --- | --- |
|  | **P-cases** | **Non-P-cases** | ***P*-value** | **OR (95%-CI)** | ***P*-value** |
|  | **2,406** | **55,822** |  |  |  |
| **N patients administered 4DSQ** | **6 (0.2%)** | **79 (0.1%)** | **0.175** | **1.0 (0.4-2.5)** | **0.916** |
| Median N 4DSQ administrations per patient (IQR) | 1 (1-1) | 1 (1-1) | 0.575 |  |  |
| Median distress score 4DSQ (IQR) | 21 (15-26) | 17 (9-25) | 0.277 |  |  |
| Median depression score 4DSQ (IQR) | 4 (1-6) | 2 (0-4) | 0.437 |  |  |
| Median anxiety score 4DSQ (IQR) | 4 (2-14) | 3 (0-8) | 0.249 |  |  |
| Median somatization score 4DSQ (IQR) | 17 (9-20) | 11 (5-16) | 0.144 |  |  |
| **N patients administered BDI2** | **28 (1.2%)** | **195 (0.3%)** | **<0.05** | **3.3 (2.2-5.0)** | **<0.05** |
| Median N BDI2 administrations per patient (IQR) | 1 (1-1) | 1 (1-1) | 0.541 |  |  |
| Median score BDI2 (IQR) | 25 (16-35) | 19 (12-31) | 0.153 |  |  |
| OR=odds ratio, 95%-CI=95% confidence interval  † Logistic regression analysis with being a ‘P-case’ as dependent variable and ≥1 4DSQ administrations and ≥1 BDI2 administrations as dichotomous independent variables | | | | | |

Table 3. Morbidity before 2014

|  |  |  | **Bivariate analysis** | **Logistic regression†** | |
| --- | --- | --- | --- | --- | --- |
|  | **P-cases** | **Non-P-cases** | ***P*-value** | **OR (95%-CI)** | ***P*-value** |
|  | **2,406** | **55,822** |  |  |  |
| **Cancer** | ***N*=133 (5.5%)** | ***N*=3,206 (5.7%)** | **0.656** | **0.9 (0.7-1.1)** | **0.248** |
| Median N years per episode (IQR) | 5.7 (2.2-10.5) | 6.9 (2.9-10.5) | 0.218 |  |  |
| **Cardiovascular disease** | ***N*=361 (15.0%)** | ***N*=8,195 (14.7%)** | **0.661** | **0.9 (0.8-1.0)** | **0.075** |
| Median N years per episode (IQR) | 8.3 (4.2-10.5) | 8.6 (4.5-10.5) | 0.349 |  |  |
| **Fatigue/sleeping disorders** | ***N*=386 (16.0%)** | ***N*=5,634 (10.1%)** | **<0.05** | **1.6 (1.4-1.8)** | **<0.05** |
| Median N years per episode (IQR) | 0.5 (0.0-2.2) | 0.3 (0.0-2.1) | <0.05 |  |  |
| **Irritable bowel disease** | ***N*=14 (0.6%)** | ***N*=386 (0.7%)** | **0.524** | **0.8 (0.4-1.3)** | **0.307** |
| Median N years per episode (IQR) | 9.5 (3.4-10.5) | 9.2 (3.6-10.5) | 0.915 |  |  |
| **Metabolic/eating disorders** | ***N*=226 (9.4%)** | ***N*=4,412 (7.9%)** | **<0.05** | **1.1 (1.0-1.3)** | **0.082** |
| Median N years per episode (IQR) | 5.2 (1.5-10.3) | 6.0 (2.1-10.5) | 0.234 |  |  |
| **Neurological disorders** | ***N*=178 (7.4%)** | ***N*=2,616 (4.7%)** | **<0.05** | **1.5 (1.3-1.8)** | **<0.05** |
| Median N years per episode (IQR) | 6.7 (2.5-10.5) | 6.8 (3.0-10.5) | 0.552 |  |  |
| **Respiratory disorders** | ***N*=145 (6.0%)** | ***N*=3,012 (5.4%)** | **0.181** | **1.0 (0.8-1.2)** | **0.933** |
| Median N years per episode (IQR) | 8.8 (3.8-10.5) | 8.7 (4.2-10.5) | 0.523 |  |  |
| **Rheumatic disorders** | ***N*=46 (1.9%)** | ***N*=667 (1.2%)** | **<0.05** | **1.5 (1.1-2.0)** | **<0.05** |
| Median N years per episode (IQR) | 6.4 (3.0-10.5) | 8.0 (3.8-10.5) | 0.349 |  |  |
| **Substance abuse** | ***N*=203 (8.4%)** | ***N*=3,133 (5.6%)** | **<0.05** | **1.5 (1.3-1.7)** | **<0.05** |
| Median N years per episode (IQR) | 3.0 (1.1-8.3) | 4.2 (1.4-8.8) | 0.089 |  |  |
| OR=odds ratio, 95%-CI=95% confidence interval  The median N episodes per patient was 1 for all listed conditions for both groups (IQR 1-1)  † Logistic regression analysis with being a ‘P-case’ as dependent variable and having ≥1 ICPC codes from the separate condition clusters as dichotomous independent variables. Table 1, Appendix provides an overview of the ICPC codes in the separate condition clusters | | | | | |

Table 4. Nervous system medication before 2014

|  |  |  | **Bivariate analysis** | **Logistic regression†** | |
| --- | --- | --- | --- | --- | --- |
|  | **P-cases** | **Non-P-cases** | ***P*-value** | **OR (95%-CI)** | **P-value** |
|  | **2,406** | **55,822** |  |  |  |
| **General anaesthetics (N01A)*** | ***N*=1 (0.0%)** | ***N*=1 (0.0%)** | **<0.05** |  |  |
| Median N prescriptions per patient | 1 (1-1) | 1 (1-1) | 1.000 |  |  |
| Median N days per prescription | 0 (0-0) | 90 (90-90) | 1.000 |  |  |
| **Local anaesthetics (N01B)** | ***N*=136 (5.7%)** | ***N*=2,859 (5.1%)** | **0.248** | **0.9 (0.8-1.1)** | **0.469** |
| Median N prescriptions per patient (IQR) | 1 (1-2) | 1 (1-1) | <0.05 |  |  |
| Median N days per prescription (IQR) | 10 (5-30) | 10 (5-30) | 0.745 |  |  |
| **Opioids (N02A)** | ***N*=253 (10.5%)** | ***N*=3,549 (6.4%)** | **<0.05** | **1.3 (1.1-1.5)** | **<0.05** |
| Median N prescriptions per patient (IQR) | 1 (1-3) | 1 (1-3) | 0.786 |  |  |
| Median N days per prescription (IQR) | 10 (9-20) | 11 (9-20) | 0.887 |  |  |
| **Other analgesics and antipyretics (N02B)** | ***N*=243 (10.1%)** | ***N*=4,175 (7.5%)** | **<0.05** | **1.0 (0.9-1.2)** | **0.903** |
| Median N prescriptions per patient (IQR) | 1 (1-3) | 1 (1-2) | <0.05 |  |  |
| Median N days per prescription (IQR) | 10 (7-19) | 10 (7-16) | 0.261 |  |  |
| **Antimigraine preparations (N02C)** | ***N*=98 (4.1%)** | ***N*=1,241 (2.2%)** | **<0.05** | **1.5 (1.2-1.9)** | **<0.05** |
| Median N prescriptions per patient (IQR) | 3 (1-10) | 2 (1-7) | 0.172 |  |  |
| Median N days per prescription (IQR) | 12 (4-31) | 12 (6-30) | 0.769 |  |  |
| **Antiepileptics (N03A)** | ***N*=54 (2.2%)** | ***N*=794 (1.4%)** | **<0.05** | **0.9 (0.7-1.3)** | **0.726** |
| Median N prescriptions per patient (IQR) | 2 (1-7) | 2 (1-5) | 0.940 |  |  |
| Median N days per prescription (IQR) | 81 (18-91) | 52 (16-97) | 0.850 |  |  |
| **Anticholinergic agents (N04A)*** | ***N*=0 (0.0%)** | ***N*=9 (0.0%)** | **0.533** |  |  |
| Median N prescriptions per patient (IQR) |  | 2 (1-5) |  |  |  |
| Median N days per prescription (IQR) |  | 52 (19-104) |  |  |  |
| **Dopaminergic agents (N04B)** | ***N*=18 (0.7%)** | ***N*=184 (0.3%)** | **<0.05** | **1.4 (0.9-2.4)** | **0.164** |
| Median N prescriptions per patient (IQR) | 1 (1-2) | 2 (1-4) | 0.060 |  |  |
| Median N days per prescription (IQR) | 25 (13-86) | 45 (15-104) | 0.152 |  |  |
| **Antipsychotics (N05A)** | ***N*=23 (1.0%)** | ***N*=195 (0.3%)** | **<0.05** | **1.7 (1.1-2.6)** | **<0.05** |
| Median N prescriptions per patient (IQR) | 2 (1-5) | 1 (1-3) | 0.480 |  |  |
| Median N days per prescription (IQR) | 59 (30-126) | 33 (15-90) | 0.071 |  |  |
| **Anxiolytics (N05B)** | ***N*=284 (11.8%)** | ***N*=3,666 (6.6%)** | **<0.05** | **1.4 (1.3-1.7)** | **<0.05** |
| Median N prescriptions per patient (IQR) | 1 (1-4) | 1 (1-2) | <0.05 |  |  |
| Median N days per prescription (IQR) | 12 (8-23) | 10 (7-18) | <0.05 |  |  |
| **Hypnotics and sedatives (N05C)** | ***N*=237 (9.9%)** | ***N*=2,944 (5.3%)** | **<0.05** | **1.4 (1.2-1.7)** | **<0.05** |
| Median N prescriptions per patient (IQR) | 1 (1-3) | 1 (1-4) | 0.845 |  |  |
| Median N days per prescription (IQR) | 19 (10-30) | 20 (10-30) | 0.089 |  |  |
| **Antidepressants (N06A)** | ***N*=132 (5.5%)** | ***N*=1,245 (2.2%)** | **<0.05** | **1.7 (1.4-2.1)** | **<0.05** |
| Median N prescriptions per patient (IQR) | 2 (1-5) | 2 (1-3) | 0.394 |  |  |
| Median N days per prescription (IQR) | 53 (23-118) | 44 (25-98) | 0.490 |  |  |
| **Psychostimulants (N06B)** | ***N*=12 (0.5%)** | ***N*=256 (0.5%)** | **0.776** | **1.0 (0.5-1.8)** | **0.953** |
| Median N prescriptions per patient (IQR) | 2 (1-3) | 3 (1-6) | 0.331 |  |  |
| Median N days per prescription (IQR) | 90 (31-103) | 49 (30-90) | 0.139 |  |  |
| **Anti-dementia drugs (N06D)** | ***N*=12 (0.5%)** | ***N*=93 (0.2%)** | **<0.05** | **2.1 (1.1-3.9)** | **<0.05** |
| Median N prescriptions per patient (IQR) | 1 (1-3) | 2 (1-3) | 0.637 |  |  |
| Median N days per prescription (IQR) | 30 (9-80) | 44 (28-100) | 0.100 |  |  |
| **Parasympathomimetics (N07A)*** | ***N*=1 (0.0%)** | ***N*=8 (0.0%)** | **0.293** |  |  |
| Median N prescriptions per patient (IQR) | 17 (17-17) | 1 (1-2) | 0.222 |  |  |
| Median N days per prescription (IQR) | 90 (90-90) | 15 (11-40) | 0.222 |  |  |
| **Drugs used in addictive disorders (N07B)** | ***N*=53 (2.2%)** | ***N*=779 (1.4%)** | **<0.05** | **1.2 (0.9-1.6)** | **0.173** |
| Median N prescriptions per patient (IQR) | 1 (1-1) | 1 (1-2) | 0.371 |  |  |
| Median N days per prescription (IQR) | 30 (18-64) | 34 (18-66) | 0.654 |  |  |
| **Anti-vertigo preparations (N07C)** | ***N*=66 (2.7%)** | ***N*=1,114 (2.0%)** | **<0.05** | **1.0 (0.8-1.3)** | **0.940** |
| Median N prescriptions per patient (IQR) | 1 (1-2) | 1 (1-2) | 0.556 |  |  |
| Median N days per prescription (IQR) | 10 (10-19) | 12 (10-27) | 0.349 |  |  |
| **Other nervous system drugs (N07X)*** | ***N*=1 (0.0%)** | ***N*=1 (0.0%)** | **<0.05** |  |  |
| Median N prescriptions per patient (IQR) | 8 (8-8) | 4 (4-4) | 1.000 |  |  |
| Median N days per prescription (IQR) | 30 (30-30) | 90 (90-90) | 1.000 |  |  |
| Unlisted ATC ‘N’ groups were not prescribed  * ATC groups were excluded from logistic regression analysis due to insufficient patients  † Logistic regression analysis with being a ‘P-case’ as dependent variable and having ≥1 prescriptions from the separate ATC codes as dichotomous independent variables | | | | | |

Table 5. Treatments/ referrals before 2014

|  |  |  | **Bivariate analysis** | **Logistic regression†** | |
| --- | --- | --- | --- | --- | --- |
|  | **P-cases** | **Non-P-cases** | ***P*-value** | **OR (95%-CI)** | **P-value** |
|  | **2,406** | **55,822** |  |  |  |
| Dietary care |  |  |  |  |  |
| N patients referred | 100 (4.2%) | 2,042 (3.7%) | 0.204 | 1.0 (0.8-1.3) | 0.841 |
| Median N referrals per patient (IQR) | 1 (1-1) | 1 (1-1) | 0.592 |  |  |
| Minimal interventions |  |  |  |  |  |
| N patients referred | 128 (5.3%) | 1,199 (2.1%) | <0.05 | 2.2 (1.8-2.7) | <0.05 |
| Median N referrals per patient (IQR) | 1 (1-3) | 1 (1-2) | <0.05 |  |  |
| Physical exercise therapies |  |  |  |  |  |
| N patients referred | 456 (19.0%) | 9,092 (16.3%) | <0.05 | 1.1 (1.0-1.2) | <0.05 |
| Median N referrals per patient (IQR) | 1 (1-2) | 1 (1-2) | <0.05 |  |  |
| Psychology |  |  |  |  |  |
| N patients referred | 192 (8.0%) | 2,649 (4.7%) | <0.05 | 1.5 (1.3-1.7) | <0.05 |
| Median N referrals per patient (IQR) | 1 (1-1) | 1 (1-1) | 0.368 |  |  |
| Psychiatry |  |  |  |  |  |
| N patients referred | 53 (2.2%) | 626 (1.1%) | <0.05 | 1.6 (1.2-2.1) | <0.05 |
| Median N referrals per patient (IQR) | 1 (1-1) | 1 (1-1) | 0.960 |  |  |
| Psychosocial care |  |  |  |  |  |
| N patients referred | 22 (0.9%) | 191 (0.3%) | <0.05 | 2.1 (1.4-3.3) | <0.05 |
| Median N referrals per patient (IQR) | 1 (1-2) | 1 (1-1) | 0.126 |  |  |
| † Logistic regression analysis with being a ‘P-case’ as dependent variable and having ≥1 of the separate treatments/referrals as dichotomous independent variables. The separate treatments/ referrals are explained in the methods | | | | | |

Table 6. Consumption of GP services before 2014

|  |  |  | **Bivariate analysis** | **Logistic regression†** | |
| --- | --- | --- | --- | --- | --- |
|  | **P-cases** | **Non-P-cases** | ***P*-value** | **OR (95%-CI)** | **P-value** |
|  | **2,406** | **55,822** |  |  |  |
| **Consultations** | ***N*=1,650 (68.6%)** | ***N*=44,362 (79.5%)** | **<0.05** |  |  |
| Median N per patient (IQR) | 15 (7-27) | 11 (5-21) | <0.05 |  |  |
| Median N per patient per year (IQR) | 2 (2-4) | 2 (1-3) | <0.05 | 0.96 (0.93-0.99) | <0.05 |
| **Double consultations** | ***N*=1,386 (57.6%)** | ***N*=30,521 (54.7%)** | **<0.05** |  |  |
| Median N per patient (IQR) | 3 (2-7) | 2 (1-5) | <0.05 |  |  |
| Median N per patient per year (IQR) | 1 (1-2) | 1 (1-2) | <0.05 | 1.19 (1.14-1.24) | <0.05 |
| **Telephone consultations** | ***N*=1,460 (60.7%)** | ***N*=35,559 (63.7%)** | **<0.05** |  |  |
| Median N per patient (IQR) | 5 (2-9) | 3 (2-6) | <0.05 |  |  |
| Median N per patient per year (IQR) | 2 (1-2) | 1 (1-2) | <0.05 | 1.06 (1.02-1.10) | <0.05 |
| **Home visits** | ***N*=215 (8.9%)** | ***N*=3,984 (7.1%)** | **<0.05** |  |  |
| Median N per patient (IQR) | 1 (1-4) | 1 (1-2) | <0.05 |  |  |
| Median N per patient per year (IQR) | 1 (1-2) | 1 (1-1) | <0.05 | 1.14 (1.06-1.23) | <0.05 |
| † Logistic regression analysis with being a ‘P-case’ as dependent variable and the median N per patient per year as continuous independent variables | | | | | |
